# Ni Foam-Supported Tin Oxide Nanowall Array: An Integrated Supercapacitor Anode

**DOI:** 10.3390/molecules26154517

**Published:** 2021-07-27

**Authors:** Ye Tian, Qi Wang, Zhijian Peng, Shundong Guan, Xiuli Fu

**Affiliations:** 1 School of Science, China University of Geosciences, Beijing 100083, China; cugbtianye@gmail.com (Y.T.); wangqi@xpu.edu.cn (Q.W.); 2 School of Science, Beijing University of Posts and Telecommunications, Beijing 100876, China; sdguan@mail.tsinghua.edu.cn

**Keywords:** integrated anode, supercapacitor, nanowall array, oxygen-deficient tin oxide, Ni-Sn alloys

## Abstract

A novel product consisting of a homogeneous tin oxide nanowall array with abundant oxygen deficiencies and partial Ni-Sn alloying onto a Ni foam substrate was successfully prepared using a facile solvothermal synthesis process with subsequent thermal treatment in a reductive atmosphere. Such a product could be directly used as integrated anodes for supercapacitors, which showed outstanding electrochemical properties with a maximum specific capacitance of 31.50 mAh·g^−1^ at 0.1 A·g^−1^, as well as good cycling performance, with a 1.35-fold increase in capacitance after 10,000 cycles. An asymmetric supercapacitor composed of the obtained product as the anode and activated carbon as the cathode was shown to achieve a high potential window of 1.4 V. The excellent electrochemical performance of the obtained product is mainly ascribed to the hierarchical structure provided by the integrated, vertically grown nanowall array on 3D Ni foam, the existence of oxygen deficiency and the formation of Ni-Sn alloys in the nanostructures. This work provides a general strategy for preparing other high-performance metal oxide electrodes for electrochemical applications.

## 1. Introduction

Nowadays, the rapid development of industry and technology is accompanied by the increasing consumption of energy [1]. However, the depletion of the most widely-used fossil fuel and the resultant environmental pollution have compelled scientists to carry out research on using renewable resources to produce energy [2]. Among them, electrochemical energy-storing devices of high energy as well as power density have attracted great attention in recent years [3]. Supercapacitors, also called electrochemical capacitors or ultracapacitors, have been widely regarded as a kind of necessary and promising energy storage device in the recent decade because of their light weight, high specific power, broad operating temperature range, ultrafast charging-discharging rate, long cyclic stability and superior reversibility [4,5]. Generally, three types of supercapacitor electrodes are classified based on their charge storage mechanisms, namely electrochemical double-layer (EDL) electrodes, pseudo-capacitive (PC) electrodes, and battery-type electrodes [6,7,8]. EDL electrodes store charge by electrostatic adsorption/desorption of ions at interfaces between the electrodes and the electrolytes, in which no chemical reaction is involved in the charging–discharging process, while PC electrodes work by rapid and reversible faradic reactions on the electrode surface for energy storage [6]. For example, carbon-based substances such as carbon nanofibers, activated carbon, carbon aerogel as well as graphene have been used as EDL electrodes; however, they can only deliver relatively limited specific capacitance, which makes them unsuitable as high-density energy-storage devices. Instead, PC electrodes, which are comprised of various transition metal oxides, metal chalcogenides, metal carbide and nitrides and conducting polymers as active electrode materials with much higher specific capacitance, have been intensively developed and widely used. Although PC electrodes have the advantages of large specific capacitance and power density together with long cycling life, the main drawback for broad applications in practice is their intrinsically inferior energy density compared with EDL electrodes [9,10]. In contrast to ordinary supercapacitors based on capacitive (EDL and PC) electrodes, where the energy-storage mechanism is on and/or near the surface, battery-type electrodes store charges throughout the entire electrodes via the complete faradaic reactions, but their cycling stability is poor [11,12]. Therefore, the development of new supercapacitors with even higher performance has been much desired [13].

Moreover, compared with currently widely-used lithium-ion batteries, the disadvantage of low energy density for supercapacitors limits their practical applications in high-power devices. For supercapacitors, the energy density (E) depends on the device capacitance (C) and operating voltage (V) of the electrode material itself since E = ½ CV^2^, so it is crucial to develop a new type of high-capacity electrode material for supercapacitors [10]. As is known, active materials for high-performance supercapacitor electrodes are generally characteristic of high electrical conductivity, large theoretical capacity, high electrochemical oxidation/reduction rate and outstanding service stability. Currently, various transition metal oxides including RuO_2_, NiO, CoO and MnO_2_ as well as conductive polymers such as polyaniline and polypyrrole with various oxidation states are widely used as supercapacitor electrode materials, in which capacitances are generated by the reversible redox transition of electroactive materials [14,15]. Among them, RuO_2_, with its high electrochemical response as an electrode and high specific capacity of 720 F·g^−1^, has been regarded as the most excellent electrode material for supercapacitors [16]. However, the rarity and high cost of RuO_2_ hinders its wide application in supercapacitors [4]. Therefore, there is an urgent need for future electrochemical researchers to find alternative materials of other components to use as electrochemical electrodes.

Among all kinds of metal oxides, tin oxide is considered an ideal anode candidate material for a wide range of applications in gas sensors, solar cells, lithium-ion batteries, as well as electrocatalysts [17,18,19,20]. In particular, tin oxide is noted to be a new active electrode material to manufacture high-performance pseudocapacitors because of its low cost, non-toxicity, superior capacitive behavior and high thermal stability [10,21,22]. However, the major disadvantages of tin oxide as an active material for supercapacitors are its poor electrical conductivity, obvious agglomeration of particles and inferior rate capability, which greatly limits its commercial application as an electrode material [23]. Thus, numerous studies in the literature have concentrated on improving the electrochemical performance of tin oxide as a supercapacitor electrode material. Among them, synthesizing composite materials of tin oxide nanostructures with highly conductive carbon-containing materials is an effective approach to improving the conductivity of tin oxide electrode materials; this results in rapid charge transfer and ultimately improves the electrochemical properties of the material. For example, Selvan et al. [24] synthesized SnO_2_@C nanocomposites and measured the capacitance of electrodes, showing that the maximal specific capacitance for the fabricated nanocomposite electrodes was 37.8 F·g^−1^ measured at 5 mV·s^−1^. Hong et al. [25] proposed a SnO_2_-wrapped functionally-carbonized cotton cloth composite via solvothermal reaction and with a following calcination process, indicating that the composite could exhibit a high specific capacitance up to 197.7 F·g^−1^ measured at a current density of 1 A·g^−1^. Ramesh et al. [26] fabricated porous graphene-based composite materials of SnO_2_/N-doped graphene oxides by a thermal treatment process in the reductive atmosphere produced by ammonia as well as urea at the optimal temperature of 550 °C, reporting that the maximal specific capacitance could reach 378 F·g^−1^ measured at 4 A·g^−1^. Furthermore, composites made from a mixture of different metal oxides also exhibit significantly improved electrochemical performance. Mevada et al. [22] prepared a nanocomposite electrode by depositing ruthenium oxide onto tin oxide nanostructures via an electrodeposition method. The areal capacitance for the optimal electrode could reach 794 mF·cm^−2^ measured at 5 mV·s^−1^ in 1 M H_2_SO_4_ electrolyte. Chuai et al. [27] successfully synthesized CuO/SnO_2_ heterojunctions by using a hydrothermal reaction approach followed by thermal treatment, reporting the rate property of 1695 F·g^−1^ measured at 10 A·g^−1^. More recently, Bhaskar et al. [28] fabricated a mesoporous SnO_2_/NiO composite electrode with the highest specific capacitance, up to 464 F·g^−1^ measured at 5 mV·s^−1^, as well as outstanding capacitance retention of 87.24% after testing for 1000 cycles. Moreover, except for the various kinds of compositing, a survey of the literature also indicates that rational morphology manipulation of nanostructured electrodes is very helpful to further improve the performance of electrodes, because more sites can be created on the electrodes for their redox reactions with the electrolyte [11]. Thus, various tin oxide materials with novel nanostructures such as nanobelts, nanoflowers, nanorods, nanowires and so on have also been reported. For instance, Rani et al. [29] fabricated a surfactant-free SnO_2_ nanoplate via a simple one-pot hydrothermal route, reporting that the highest specific capacitance could reach 120.41 F·g^−1^ measured at 5 mV·s^−1^. Wang et al. [30] prepared a nanorod array of tin oxides onto nickel (Ni) foam with a maximum specific capacitance of 101.1 mAh·g^−1^ measured at 5 A·g^−1^. Meng et al. [31] synthesized SnO_2_ nanoflowers assembled from nanopetals on Ti substrate through a facile hydrothermal approach, indicating that the capacitance of the composite was up to 437 mF·cm^−1^ measured at 25 mV·s^−1^. Among them, an independent three-dimensional framework nanostructure with a suitable porous surface is considered an ideal electrochemical electrode structure [7].

**Figure 1:**
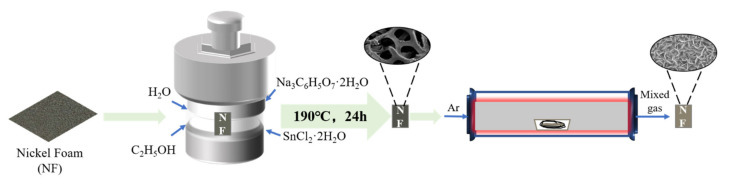
Illustration of the synthesis process for the proposed Ni@a-SnO_x_ NWs.

Furthermore, it is well-known that synthesizing electroactive materials on current collectors has been a promising strategy for preparing electrodes without binders because it can avert the complex electrode-making process for powdery samples. This strategy can also allow more active materials to directly touch the electrolyte to participate in the redox reaction, thus boosting the electrochemical properties of the electrode materials. Among various current collectors, Ni foam is noted as an ideal substrate for fabricating binder-free electrodes for PCs. Therefore, in this work, a tin oxide nanowall array onto Ni foam (Ni@SnO_x_ NWs) was deposited by a facile solvothermal reaction utilizing a specially designed process (see Figure 1). The sample was then thermally treated in a safe, environmentally-friendly and reductive atmosphere provided by the pyrolysis of preoxidized polyacrylonitrile (PAN) powder, finally obtaining an interesting product of Ni foam-supported, partly Ni-Sn-alloyed and oxygen-deficient tin oxide nanowall array (Ni@a-SnO_x_ NWs). This product can be directly used as the electrode for supercapacitors and shows greatly improved electrochemical performance, with a maximum specific capacitance of 31.50 mAh·g^−1^ measured at 0.1 A·g^−1^, as well as outstanding cycling performance, with a 1.35 times increase in capacitance after 10,000 cycles. Furthermore, an asymmetric supercapacitor (ASC) composed of the obtained products as the anode and activated carbon (AC) as the cathode can achieve a high potential window of 1.4 V. In such a product, the nanowall arrays of the electrode material could not only shorten the ion diffusion route between the electrode and electrolyte, but also enhance the area of highly-activated surface and offer abundant electroactive sites for redox reactions [32,33]. Ni-Sn alloys can function as the active materials of electrodes as well for battery-type characteristic supercapacitors with outstanding electrical conductivity and superior electrochemical properties. Additionally, the presence of tin oxides with oxygen deficiency can enhance electrical performance in two ways: (a) the oxygen-deficient metal oxides exhibit better electrochemical performance and cyclic stability than stoichiometric meal oxides, possibly because the presence of oxygen vacancy could act as shallow donors, improving conductivity of the electrode materials as well as the wettability and exposing more active sites [29,30,31]; and (b) the abundant oxidation states in oxygen-deficient metal oxides are conducive to rapid and reversible redox reactions, thereby contributing to the high theoretical capacities of electrodes [34]. This work will provide a general strategy for preparing other high-performance metal oxide electrodes for electrochemical applications.

## 2. Experimental

### 2.1. Chemicals and Materials

Trisodium citrate dehydrate (Na_3_C_6_H_5_O_7_·2H_2_O, 99.0%), stannous chloride (SnCl_2_·2H_2_O, >98.0%), AC powder, acetylene black (ACET), polytetrafluoroethylene (PTFE) and absolute ethanol were commercially available from Aladdin Industrial Corporation (Shanghai, China). These chemicals were used directly without further purification. Ni foams with a thickness of 0.3 mm were supplied by Changde Liyuan New Materials Co. LTD (Changde, China). Before use, foams were ultrasonically cleaned with absolute ethanol and deionized water in turn, and thereafter dried at about 100 °C in an open oven. In addition, PAN powder was purchased from Sinopharm Chemical Reagent Co., Ltd. (Shanghai, China).

### 2.2. Synthesis of Ni@SnO_x_ NWs

SnO_x_ NWs were grown onto Ni foam surface by a solvothermal synthesis method as follows [28]. Firstly, 40 mL deionized water was filled in a beaker, and 2.94 g Na_3_C_6_H_5_O_7_·2H_2_O and 2.04 g SnCl_2_·2H_2_O were then dissolved in the water under strong magnetic stirring for 30 min in air at about 25 °C, obtaining a homogeneous solution. Afterwards, 40 mL absolute ethanol was slowly added into the beaker, continuing magnetic stirring for 30 min again, obtaining a slightly white turbid precursor solution. The precursor solution was subsequently fed in an autoclave (100 mL), and a cleaned Ni foam wafer (4 cm × 4 cm) was completely immersed in the solution vertically. Afterwards, the sealed autoclave was moved into an electric oven and heated at 180 °C for 12 h. Finally, after natural cooling to ambient temperature, the Ni foam was collected from the autoclave, rinsed with deionized water and absolute ethanol for 3 times, and fully dried at approximately 60 °C in air for 10 h. The finally obtained sample was defined as Ni@SnO_x_ NWs.

### 2.3. Synthesis of Ni@a-SnO_x_ NWs

The obtained Ni@SnO_x_ NWs sample was first cut into smaller wafers (1 cm × 1.5 cm), placed at the bottom of an alumina crucible, and surrounded by 4 g of the PAN powder. Subsequently, the crucible was set half-opened and placed at the center of a tube furnace. After fully evacuating the air in the furnace tube, the samples were calcined at 425 °C for 150 min in high-purity Ar flow (100 sccm). The proposed Ni@a-SnO_x_ NWs samples were eventually obtained after natural cooling down to ambient temperature.

### 2.4. Samples Characterization

The loading mass of active materials on each electrode was measured on the basis of the mass change of the Ni foams before and after sample preparation. A scanning electron microscope (SEM, Gemini SEM 500, Düsseldorf, Germany) was utilized to examine the microstructures and morphologies of the prepared samples. An X-ray diffractometer (XRD, D/max-2500/PC, Tokyo, Japan) was applied to determine their phase composition and crystal structure. The XRD tests were conducted in continuous scanning mode at the speed of 5°/min using copper Kα radiation with a wavelength of 1.5418 Å. Their chemical state and surface elemental composition were characterized by a Thermo Fisher X-ray photoelectron spectroscope (XPS, Waltham, MA, USA) using nonmonochromated aluminum Kα radiation with a photon energy of 1486.7 eV. And the correction of the energy shift was executed using carbon 1s line with the binding energy of 284.6 eV as reference [23].

### 2.5. Electrochemical Evaluation

An electrochemical workshop (CHI660E, Chenhua Instruments, Shanghai, China) was used to record the cyclic voltammetry (CV) and galvanostatic charge–discharge (GCD) plots of the samples. Their electrochemical capacitive performances were examined by using a three-electrode system within 6 M KOH aqueous electrolyte solution. Their cycling stabilities were investigated by a Land CT3001A battery test kit (Wuhan Lanhe Electronics Ltd., Wuhan, China). For the measurements, the obtained products were cropped into 1 × 2 cm^2^ square pieces directly for working electrodes, which were immersed in electrolyte with a reaction area of 1 × 1 cm^2^. Meanwhile, the three electrode system contained Pt foil as the counter electrode as well as Hg/HgO electrode as the reference electrode. The ASC cell was constructed with the as-prepared Ni@a-SnO_x_ NWs samples as the anode, AC as the cathode, glass fiber film as the separator and 6 M KOH as the electrolyte. The cathode was prepared by pasting the mixture of AC (85 wt%) with acetylene black (10 wt%) and PTFE (5 wt%) on a piece of nickel foam and then pressing it into a sheet at 10 MPa pressure for 30 s. The performance of the ASC cell was also examined by the CHI660E electrochemical workshop and Land test system with a two-electrode kit. All electrochemical tests were executed at 25 °C.

On the basis of the collected GCD data on the obtained electrodes, their specific capacitances (*C_s_*, mAh·g^−1^), mass energy density (*E*, Wh·kg^−1^) and power density (*P*, W·kg^−1^) could be calculated through the following Equation [5]:

(1) $$C_{s}=\frac{I\times \Delta t}{3.6\times m}$$

(2) $$E=\frac{I\times \int Vdt}{3.6\times m}$$

(3) $$P=\frac{E\times 3600}{\Delta t}$$

in which *I* is the discharging current (A), ∆*t* represents the discharging time (s) and *m* expresses the mass of the active materials on the working electrodes (g), and ∫*Vdt* is the integral area under the discharge curve, respectively. In this work, the mass of the active materials grown onto the Ni foam was measured as about 4 mg, and the mass of AC loaded on the cathode was measured as about 3 mg.

## 3. Results and Discussion

### 3.1. Composition, Morphology and Microstructure

**Figure 2:**
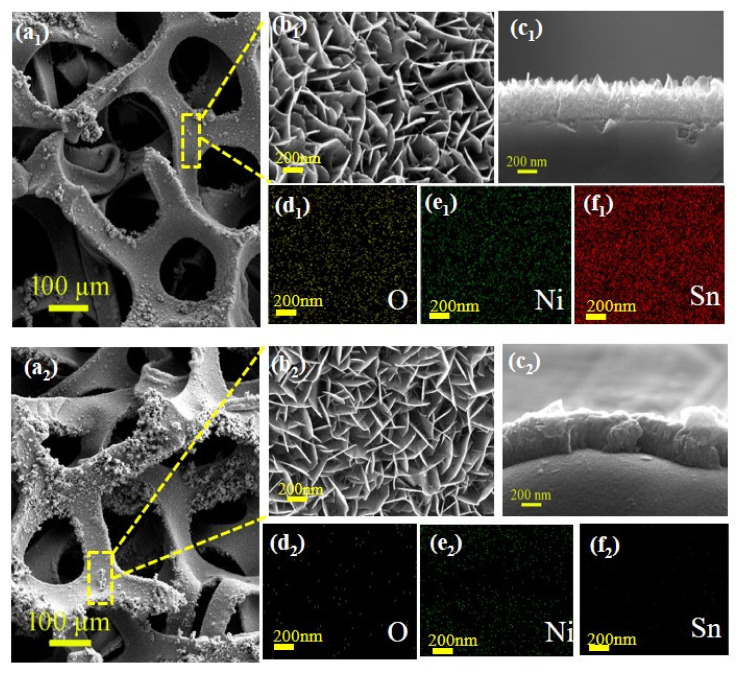
Typical SEM images of the obtained Ni@a-SnO_x_ NWs (marked as 1) and Ni@SnO_x_ NWs (marked as 2) samples in low magnification (**a_1_**,**a_2_**), in high magnification (**b_1_**,**b_2_**) and from cross-section (**c_1_**,**c_2_**), and their corresponding EDX mapping images of (**d_1_**,**d_2_**) O, (**e_1_**,**e_2_**) Ni, (**f_1_**,**f_2_**) Sn.

In the present work, a simple two-step approach was proposed to fabricate the target sample (see Figure 1). First, a convenient solvothermal synthesis process using Na_3_C_6_H_5_O_7_·2H_2_O and SnCl_2_·2H_2_O as the sources was applied to grow SnO_x_ nanowall arrays onto the surface of Ni foam (Ni@SnO_x_ NWs) [28]. Then, the as-prepared Ni@SnO_x_ NWs sample was thermally treated in a reductive atmosphere containing CO, NH_3_, CH_4_ and so on produced by the pyrolysis of PAN powder to prepare the proposed Ni@a-SnO_x_ NWs sample. Figure 2a–c shows typical SEM images from the vertical and cross-sectional view of the Ni@a-SnO_x_ NWs sample in comparison with those of its Ni@SnO_x_ NWs counterpart. As shown in Figure 2a_1_,a_2_, the low-magnification images of both samples show a homogeneous, complete nanowall array over the entire area on the Ni foam framework, indicating that both the applied solvothermal synthesis and subsequent thermal treatment present no adverse effect on maintaining the framework structure of the Ni foams. In Figure 2b_1_,b_2_ from a high magnification view, it is seen that a mass of continuous, highly ordered nanosheets were vertically grown on the Ni foam substrate as if they were “standing up” and surrounding each other to form a nanoscaled wall. The erect nanowalls were interconnected to form open channels throughout the interior of the active materials, which can facilitate the entry of electrolyte solution and accelerate ion exchange and electron transfer. Furthermore, from the cross-sections as exhibited in Figure 2c_1_,c_2_, it is obviously seen that the nanowall arrays in both samples are vertically grown onto the surface of their Ni foam substrates. More importantly, compared with the clear boundary between the nanowalls and the substrate of the Ni@SnO_x_ NWs sample as displayed in Figure 2c_2_, the fuzzy boundary between them in the Ni@a-SnO_x_ NWs sample as shown in Figure 2c_1_ also demonstrates that Ni foam has taken part in the reaction with the surrounding tin oxide grains during the designed thermal treatment process, forming a Ni-Sn alloy layer at the contact interface between the tin oxide nanowalls and the Ni foam current collector in the proposed Ni@a-SnO_x_ NWs sample. Additionally, from their corresponding EDX mapping images, it can be seen that, after thermal treatment, the elemental distributions in the Ni@a-SnO_x_ NWs sample become more homogeneous.

**Figure 3:**
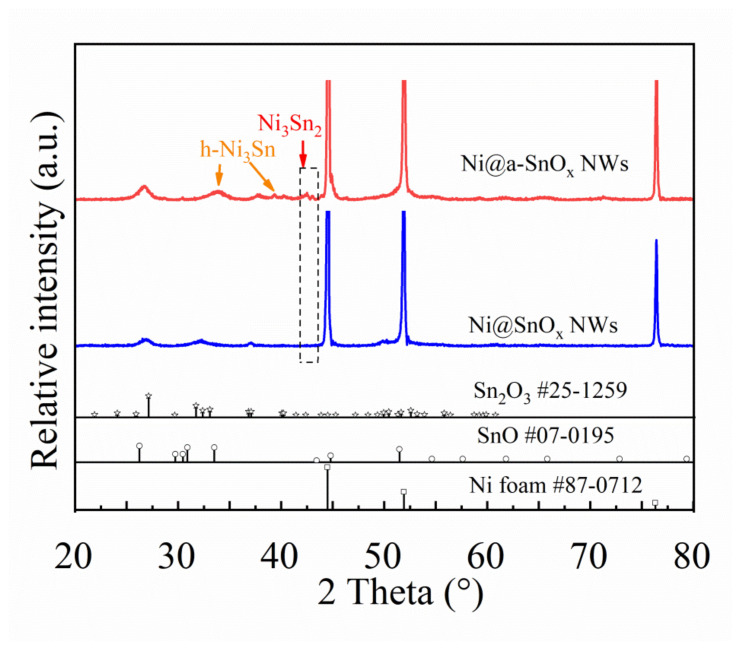
XRD pattern of the Ni@a-SnO_x_ NWs sample in comparison with that of the Ni@SnO_x_ NWs sample.

Figure 3 compares the XRD pattern of the Ni@a-SnO_x_ NWs specimen with that of its unreduced Ni@SnO_x_ NWs counterpart. As is seen, both samples exhibit strong diffraction peaks at approximately 44.4°, 51.8° and 76.3°, corresponding to the (111), (200) as well as (220) crystalline planes of the Ni phase in the samples (JCPDS card of 87-0712), respectively, proving the existing of Ni foam in them. From the pattern of the Ni@SnO_x_ NWs sample, the observed diffraction peaks at about 27.0°, 33.0° and 36.7° are well-indexed to the (011), (030) and (130) crystal planes of the Sn_2_O_3_ phase (JCPDS card of 25-1259), while partial weak peaks at roughly 61.8°, 65.8° and 72.8° are attributed to the system of SnO (JCPDS card of 07-0195). This result suggests that oxygen-deficient tin oxides have been successfully grown onto Ni foam substrates by the applied solvothermal process. Furthermore, it should be noted that no other peaks of Ni-containing impurity phases were observed in this sample. However, from the XRD pattern of the present Ni@a-SnO_x_ NWs sample, both metallic nickel and tin oxide phases could be identified as in the Ni@SnO_x_ NWs sample. In the pattern, the newly-appeared diffraction peaks at about 33.8° and 39.3° correspond to the (110) and (200) crystal planes of h-Ni_3_Sn (JCPDS card of 35-1362), and those at 43.6° and 44.7° could be identified as the (102) and (110) crystalline planes of the Ni_3_Sn_2_ phase (JCPDS card of 06-0414), indicating that Ni-Sn alloys are introduced by the chemical reactions between tin oxide nanowalls and the Ni foam substrate through the proposed thermal treatment method. Therefore, the fuzzy interface in Figure 2c_1_ may be caused by the Ni-Sn alloying. In other word, the Ni@a-SnO_x_ NWs sample may have better electrical conductivity due to the stronger bond between tin oxide nanowalls and the Ni foam substrate, as well as the presence of the Ni-Sn alloy phase, which is conducive to the electron transport in it [7].

**Figure 4:**
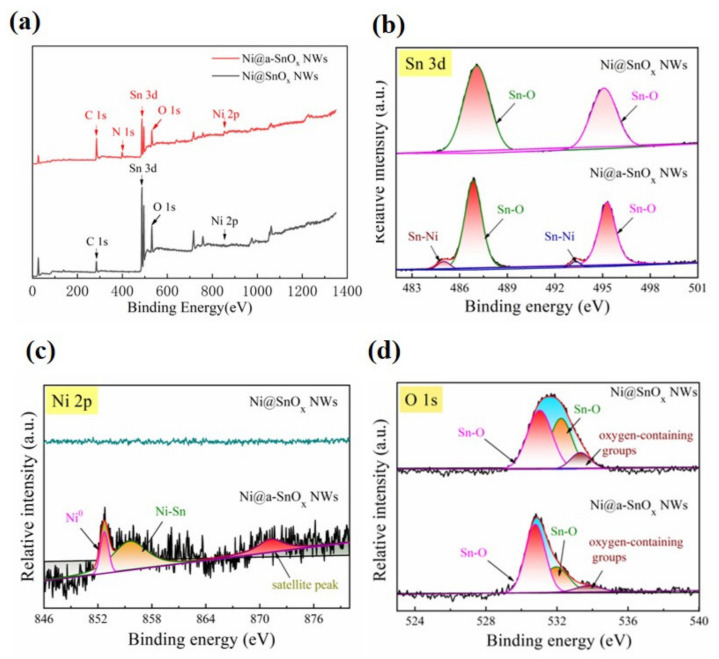
XPS data: (**a**) survey spectrum and high-resolution spectra of (**b**) Sn 3d, (**c**) Ni 2p and (**d**) O 1s of the Ni@a-SnO_x_ NWs sample. Comparatively, those of the Ni@SnO_x_ NWs sample are also presented.

To further investigate the chemical states and elemental compositions of the acquired specimens, XPS analysis was executed. The corresponding XPS full survey spectrum as well as high-resolution narrow spectra of Sn 3d, Ni 2p and O 1s of the present Ni@a-SnO_x_ NWs sample are displayed in Figure 4a–d, respectively, in comparison with those of the Ni@SnO_x_ NWs sample. From the survey spectra (Figure 4a), the existence of Sn, Ni and O elements can be confirmed. With respect to the Sn 3d spectrum of the Ni@SnO_x_ NWs sample, two symmetric peaks centered at about 487.35 and 495.37 eV could be fitted (see Figure 4b), which are correlated with Sn 3d_3/2_ as well as Sn 3d_5/2_, respectively. Such results are identical to the data for Sn 3d in SnO_x_ (Sn^2+^ or Sn^4+^) nanowall array [35]. Nevertheless, from the Sn 3d spectrum of the present Ni@a-SnO_x_ NWs sample, except for the peaks at about 486.86 and 495.28 eV assigning to SnO_x_, two other peaks centered at approximately 485.01 and 493.31 eV are observed. These results confirm the Sn-Ni bonds in Ni-Sn alloy phases such as h-Ni_3_Sn and Ni_3_Sn_2_ in the interface layer between the Ni foam substrate and tin oxide nanowall array, which is also consistent with its Ni 2p spectrum (see Figure 4c). As is seen in Figure 4c, no peaks could be identified from the Ni 2p spectrum of the Ni@SnO_x_ NWs sample, while three peaks at roughly 852.71, 855.53 and 871.20 eV of Ni 2p_3/2_ were fitted based on the recorded spectrum for the present Ni@a-SnO_x_ NWs sample. The binding energy at 855.53 eV of its Ni 2p spectrum may be ascribed to the formation of Ni-Sn alloys, while that at 852.71 eV may be assigned to metallic Ni in the Ni foam of the specimen. In addition, the peak at 871.20 eV could be considered as a satellite peak. In Figure 4d, the spectra of O 1s for both specimens can be fitted into three distinct peaks, which are in accordance with the Sn-O bonds of tin oxides and the adsorbed, oxygen-containing groups such as H_2_O on the surface of the specimens, respectively. Among them, the peaks at about 531.02 eV of the Ni@SnO_x_ NWs sample and roughly 530.78 eV of the Ni@a-SnO_x_ NWs sample are ascribed to the oxygen-deficient tin oxides, confirming the production of oxygen vacancies in the samples during the designed synthesis processes. Oxygen-deficient tin oxides can also contribute to improved conductivity for the present electrode materials and significantly facilitate surface redox reaction kinetics, thus enhancing the specific capacitance of the samples [30,31].

### 3.2. Electrochemical Performance

**Figure 5:**
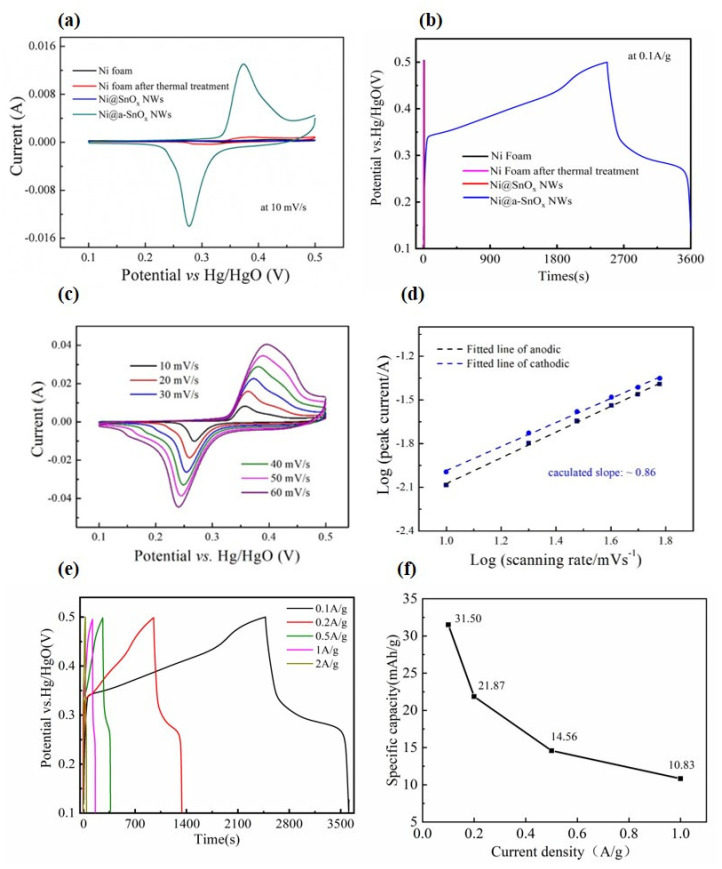
CV curves (**a**) and GCD curves (**b**) of the Ni@a-SnO_x_ NWs sample, in comparison with those of Ni foam, Ni foam after thermal treatment and the Ni@SnO_x_ NWs samples. (**c**) CV curves of the Ni@a-SnO_x_ NWs sample at different scanning rates and (**d**) linear fitting of the log(peak current) and log(scanning rate) for the anodic and cathodic peaks in the CV curves. (**e**) GCD curves of the Ni@a-SnO_x_ NWs sample at different current densities and (**f**) the corresponding specific capacitance calculated based on the GCD results.

Through using a conventional three-electrode system within 6 M NaOH aqueous electrolyte solution, the electrochemical properties of the Ni@a-SnO_x_ NWs sample as electrode materials for supercapacitors were explored. Comparatively, pure Ni foam, Ni foam after thermal treatment and the Ni@SnO_x_ NWs samples were also investigated. As is seen in the recorded CV curves (see Figure 5a), the redox peaks of pure Ni foam, Ni foam after thermal treatment and the Ni@SnO_x_ NWs samples can be ignored at a scan rate of 10 mV·s^−1^. In contrast, the recorded curves of optimal Ni@a-SnO_x_ NWs electrodes obviously exhibit a couple of redox peaks, representing that they underwent typical Faradaic process. Figure 5b illustrates the GCD curves of these samples tested at an identical current density (0.1 A·g^−1^) and potential window (0.1–0.5 V). As is seen in this figure, the charge-discharge times of pure Ni foam, Ni foam after thermal treatment as well as the Ni@SnO_x_ NWs samples are negligible, indicating their inferior electrochemical energy storage capacity. However, the target Ni@a-SnO_x_ NWs sample presents a distinct charge-discharge plateau with a significantly long time, revealing that it has quite good electrochemical energy storage performance, which could be owing to the above-mentioned Ni-Sn alloying during the designed thermal treatment. As is already known in the last section, the presence of Ni-Sn alloys in the target sample can significantly improve its electrical conductivity, and facilitate electron transfer due to the stronger adhesion between tin oxide nanowalls and the Ni foam substrate. The electrochemical properties of the Ni@a-SnO_x_ NWs samples were also compared with a sample without the morphology of nanowalls obtained by the direct casting of SnO_2_ suspension into Ni foam, followed by thermal treatment in the same reduced atmosphere. It was measured that the areal capacitance of the reference sample was only 0.1 mAh·cm^−2^ at the current density of 0.1 A·cm^−2^, confirming that the morphology of nanowalls directly on the Ni collector could maximize the use of electrochemically active species to improve its electrochemical performance.

The CV curves of the Ni@a-SnO_x_ NWs sample were also measured by sweeping the voltage from 0.1 to 0.5 V at various scanning rates. The results are displayed in Figure 5c. As is seen, there are a couple of obvious redox peaks at all applied scanning rates, while no significant shape variation in the CV profiles could be noticed with increasing scanning rates, which indicates an improved mass transportation and electron conduction within the present composite electrode [25]. However, the anode and cathode peaks shifted to high and low potentials respectively with the increase of scanning rate, signifying the polarization of charges, which indicates a quasi-reversible electrochemical process in the electrodes [33]. As is known, tin oxide-based electrodes are typical pseudo-capacitive materials that can store charges by Faradaic reactions. Therefore, the electrochemical reactions of oxygen-deficient SnO_x_ will become more complex due to the introduction of an extra valence state, which may result in more charge storage routes, thus acquiring higher specific capacitances. Furthermore, the higher conductivity of the present SnO_x_ with oxygen deficiencies will accelerate the transfer of electrons through the electrodes, thus further enhancing their electrochemical performance [31]. To investigate the electrochemical kinetics of the target electrode materials, their CV curves were further analyzed. Generally, the total capacitance of the pseudocapacitive materials or battery-type electrode materials originates from two types of processes: the diffusion-controlled process and capacitive-controlled process. Figure 5d illustrates the dependence of log(peak current, *i*) on log(scanning rate, *v*) in 10–60 mV·s^−1^ at various scan rates, which identifies the charge storage mechanism of the obtained sample. As is known in Ref. [36], *i* and *v* comply with the following relation:

*i* = α*v*^β^ (4)

where α and β represent two relative constants. Approximately, when β = 0.5, the electrochemical process is diffusion-controlled, by which ions are extracted and inserted [37]; but if β = 1, it represents a behavior of pure capacitance through surface capacitive effects [38]. As is seen from Figure 5d, the fitted β value is roughly 0.86, implying that the energy storage of the Ni@a-SnO_x_ NWs electrode possesses both supercapacitor-like and battery-like features, which are caused by the tin oxides of oxygen deficiencies as well as the alloys between Ni and Sn in the electrodes, respectively.

Moreover, rate capability is of much importance in the electrochemical performances of electrodes. In this study, GCD curves were measured to examine the rate capability of the Ni@a-SnO_x_ NWs sample with respect to time in a specific potential window in the same 6 M KOH solution. In Figure 5e, the GCD curves of the Ni@a-SnO_x_ NWs sample at various current densities (0.1–2 A·g^−1^) are illustrated. In general, the charge and discharge times always decrease with the increase of current density [39]. As is seen in Figure 5e, the Ni@a-SnO_x_ NWs sample has an almost identical shape of GCD curves, indicating that the redox reaction is reversible and the material has good capacitive properties [39,40]. Moreover, the corresponding specific capacitances of the Ni@a-SnO_x_ NWs sample at various current densities were calculated from the discharge curves, and their results are illustrated in Figure 5f. Generally, the capacitance of a supercapacitor will drop with increasing current density, because the discharge process will become much quicker at a higher current density. In this work, with the increase of current density from 0.1 to 2 A·g^−1^, the calculated specific capacitance on the Ni@a-SnO_x_ NWs sample decreases constantly from 31.50 to 7.78 mAh·g^−1^. When the applied current density is lower, more sites in the electrode are activated to participate into the redox reaction. Thus, a slower discharging and discharging process would cause a higher specific capacitance [40]. In contrast, when the applied current density is higher, partial active sites in the sample may fail to participate into the charging-discharging reaction, because the ion diffusion rate is usually lower than the electron transfer rate, which will result in a shorter discharge time, finally causing a lower specific capacitance [41,42]. Therefore, the obtained Ni@a-SnO_x_ NWs sample delivers the maximal specific capacitance 31.50 mAh·g^−1^ at the definite current density 0.1 A·g^−1^.

**Figure 6:**
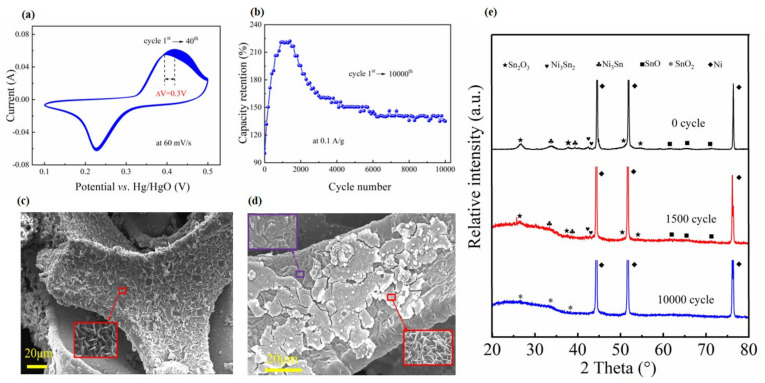
(**a**) CV Curves of the Ni@a-SnO_x_ NWs sample during 40 cyclic voltammetry cycles at a scanning rate of 60 mV·s^−1^. (**b**) Curve of the capacity retention *vs* cycle number for the Ni@a-SnO_x_ NWs sample at a current density of 0.1 A·g^−1^ during long-term GCD cycles. SEM images of the Ni@a-SnO_x_ NWs sample after 1500 charge-discharge cycles (**c**) and after 10,000 charge-discharge cycles (**d**). (**e**) XRD pattern of the Ni@a-SnO_x_ NWs sample after 1500 and 10,000 charge-discharge cycles.

Cyclic stability is one of the mandatory requirements on the electrodes of supercapacitors. To evaluate the practical performance on the present Ni@a-SnO_x_ NWs sample as supercapacitor electrodes, CV curves for the first 40 repetitive cycles and GCD curves for 10,000 repetitive cycles were measured. Figure 6a exhibits the results of the cycling tests on the present Ni@a-SnO_x_ NWs electrode, which were collected by repeatedly measuring within a selected potential window of 0.1–0.5 V at 60 mV s^−1^ for the first 40 cycles. The variation of the recorded CV curves indicated the existence of phase transformation reactions during CV scanning within the adopted potential window [7]. Figure 6b shows the curves of capacitance retention vs. cycling number at 0.1 A·g^−1^. Different from traditional electrode materials, the present Ni@a-SnO_x_ NWs sample exhibits an interesting cycling performance. That is, the special capacitance of the electrode increases dramatically in the first 1000 cycles. However, after the capacitance retention rises up to the peak at roughly the 1500th cycle, it begins to drop down sharply as the cycle further increases. However, with further cycling, the subsequently measured capacitance loss becomes much weaker, and after 6000 cycles of tests, the eventually measured specific capacitance was approximately 1.35 times that of its first cycle. It is worth noting that the capacitance of the Ni@a-SnO_x_ NWs sample shows a stable state over 6000 to 10,000 cycles. The ideal long-term cycling stability reveals that the present Ni@a-SnO_x_ NWs electrode possesses excellent electrochemical performance. The surface microstructures of the Ni@a-SnO_x_ NWs electrode samples after 1500 and 10,000 charging-discharging cycles are examined by SEM imaging. The SEM image of the Ni@a-SnO_x_ NWs sample tested for 1500 cycles is shown in Figure 6c. It is seen that its original morphology is still well maintained without the falling-off of the tin oxide nanowall array (also see the inset). However, after testing for 10,000 cycles, while the Ni foam frame is intact, part of the tin oxide nanowall array is detached from the surface of the Ni foam framework (see Figure 6d), although the residual area still maintained the initial morphology of the tin oxide nanowall array (see the inset), which presents no difference from the sample before cycling. In addition, the results of XRD analysis on the tested specimens are displayed in Figure 6e. As is seen, there is no phase change in the sample after 1500 charging-discharging cycles. However, after testing for 10,000 cycles, although the phases of metallic Ni (JCPDS card of 65-2865) and SnO_2_ (JCPDS card of 41-1445) could be detected from the pattern, the original oxygen-deficient tin oxides and Sn-Ni alloy phase disappeared, indicating that phase changes happened in the sample during the long-time cycling process. Therefore, the increase of capacity retention in the course of the initial charging-discharging process may be attributed to the long activation period of the active materials. During this period, the active substances in electrodes are activated by repeated insertion and de-insertion of ions in the electrolyte, thereby providing more active sites to participate in the redox reactions [43]. However, with regard to the prompt drop of capacity retention after 1500 cycles, it can be mainly assigned to the exfoliation of active substances (SnO_x_ and Ni-Sn alloy) from the Ni foam substrate and the collapse of nanowall alloys.

**Figure 7:**
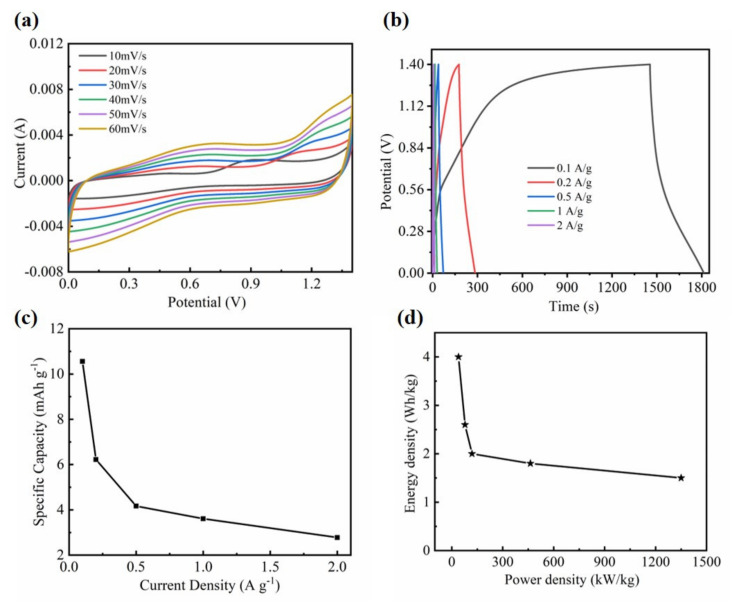
(**a**) CV curves of the as-assembled Ni@a-SnO_x_ NWs//AC ASC device at different scanning rates. (**b**) GCD curves of the Ni@a-SnO_x_ NWs//AC ASC device at various current densities. Rate performance (**c**) and Ragone plots (**d**) of the Ni@a-SnO_x_ NWs//AC ASC device.

In order to reveal the potential of practical applications of the Ni@a-SnO_x_ NWs sample, an ASC device with the optimal Ni@a-SnO_x_ NWs sample as the anode and AC as the cathode was assembled. The CV curves of the Ni@a-SnO_x_ NWs and AC electrodes were measured respectively at a sweep rate of 10 mV·s^-1^ in a three-electrode system. It was known that the potential window of Ni@a-SnO_x_ NWs was 0.1–0.5 V and that of AC was −1.0–0 V. Thus, the operating cell voltage would be extended to about 1.4 V if the two electrodes are assembled into ASC devices within the same aqueous electrolyte solution of 6.0 mol·L^-1^ KOH, which is a key factor to their increased energy density [44,45]. Figure 7a shows the CV profiles of the ASC device performed in a working potential window of 0–1.4 V at a scan rate ranging from 10 to 60 m·Vs^−1^. When the scanning rate increases from 10 to 60 mV·s^−1^, the CV curves are similar in shape, indicating the ideal fast charge-discharge characteristics of the ASC fabricated by the present Ni@a-SnO_x_ NWs electrodes [46]. GCD measurements were carried out at different current densities from 0.1 to 2 A·g^−1^ and the results are displayed in Figure 7b. The calculated specific capacitance of the ASC devices attained 10.56, 6.22, 4.17, 3.61 and 2.78 mAh·g^−1^ at 0.1, 0.2, 0.5, 1 and 2 A·g^−1^, respectively (Figure 7c). The specific capacitance of the ASC device gradually decreases with the increasing of current density, which proves its good rate capability [47]. In general, the capacitance of an ASC device is largely determined by the cell configuration and the mass of active materials in the cathode and anode. Therefore, the capacity value of a two-electrode device cannot be higher than a three-electrode system [48]. According to the above data, the energy and power density of the as-assembled ASC device are calculated. Figure 7d presents the Ragone plot of the corresponding power and energy density for the as-assembled Ni@a-SnO_x_ NWs//AC ASC device when discharging at different current densities. Based on Equations (2) and (3), the energy density of the ASC device can be estimated as 4 Wh·kg^−1^ at a power density of 39.8 kW·kg^−1^, and the energy density still retains 1.5 Wh·kg^−1^ even at a higher power density of 1350 kW·kg^−1^. In summary, our results demonstrate that the present Ni@a-SnO_x_ NWs sample, with its unique structure, high specific capacitance and outstanding stability, may be a qualified candidate as a supercapacitor electrode for future applications.

## 4. Conclusions

A novel specimen of homogeneous tin oxide nanowall array with abundant oxygen deficiencies and partial Ni-Sn alloying onto a Ni foam substrate was successfully prepared using a facile solvothermal synthesis process, subsequently followed by a safe and facile thermal treatment in an easy-controlled reductive atmosphere produced by the pyrolysis of PAN powders. The nanostructures are composed of Sn_2_O_3_, SnO as well as a Ni-Sn alloy phase, which are well adhered to the Ni foam substrate through the alloying reaction during the designed thermal treatment. When this specimen is utilized as a supercapacitor electrode, the metallic Ni foam serves as the current collector, which can improve the electrical conductivity of the specimen to enable rapid charge transfer, and the presence of oxygen deficiencies in the tin oxide nanostructures and Ni-Sn alloying can also enhance the electrical conductivity of the specimen and offer more active sites for electrochemical redox reactions, improving its electrochemical performance. Such products presented good rate capability and the recorded maximum specific capacitance reached 31.50 mAh·g^−1^ at 0.1 A·g^−1^. Additionally, different from traditional electrode materials, the specific capacitance of the optimal electrode was enhanced after a long-term charging and discharging cycling test, which could reach a 1.35-fold increase after testing for 10,000 cycles at 0.1 A·g^−1^ compared with its initial value. Finally, when the optimal specimens were directly used as integrated, binder-free electrodes for ASC, the assembled device composed of the as-obtained products as the anode and AC as the cathode achieved a high potential window of 1.4 V. This work also provides a general strategy for preparing other high-performance metal oxide electrodes for electrochemical applications.

## Data Availability

Not applicable.

## Sample Availability

Samples of the compounds in this article are available from the authors.

## References

[1] Theerthagiri J., Senthil R.A., Nithyadharseni P., Lee S.J., Durai G., Kuppusami P., Madhavan J., Choi M.Y. (2020). Recent progress and emerging challenges of transition metal sulfides based composite electrodes for electrochemical supercapacitive energy storage. Ceram. Int..

[2] Theerthagiri J., Durai G., Karuppasamy K., Arunachalam P., Elakkiya V., Kuppusami P., Maiyalagan T., Kim H.S. (2018). Recent advances in 2D nanostructured metal nitrides, carbides, and phosphides electrodes for electrochemical supercapacitors–A brief review. J. Ind. Eng. Chem..

[3] Pandit B., Jadhav C.D., Chavan P.G., Tarkas H.S., Sali J.V., Gupta R.B., Sankapal B.R. (2020). Two-dimensional hexagonal SnSe nanosheets as binder-free electrode material for high-performance supercapacitors. IEEE Trans. Power Electron..

[4] Le H.T.T., Ngo D.T., Dang V.A.D., Hoang T.T.B., Park C.J. (2020). Decoration of mesoporous carbon electrodes with tin oxide to boost their supercapacitive performance. New J. Chem..

[5] Shao Y.L., El-Kady M.F., Sun J.Y., Li Y.G., Zhang Q.H., Zhu M.F., Wang H.Z., Dunn B., Kaner R.B. (2018). Design and mechanisms of asymmetric supercapacitors. Chem. Rev..

[6] Cherusseri J., Pandey D., Sambath Kumar K., Thomas J., Zhai L. (2020). Flexible supercapacitor electrodes using metal-organic frameworks. Nanoscale.

[7] Guan S.D., Fu X.L., Zhang B., Lei M., Peng Z.J. (2020). Cation-exchange-assisted formation of NiS/SnS_2_ porous nanowalls with ultrahigh energy density for battery–supercapacitor hybrid devices. J. Mater. Chem. A.

[8] Guan S.D., Fu X.L., Lao Z.Z., Jin C.H., Peng Z.J. (2019). NiS-MoS_2_ hetero-nanosheet arrays on carbon cloth for high-performance flexible hybrid energy storage devices. ACS Sustain. Chem. Eng..

[9] Yao M., Liu A., Xing C.X., Li B.S., Pan S.S., Zhang J.H., Su P.P., Zhang H.T. (2020). Asymmetric supercapacitor comprising a core-shell TiNb_2_O_7_@MoS_2_/C anode and a high voltage ionogel electrolyte. Chem. Eng. J..

[10] Geng J.G., Ma C.T., Zhang D., Ning X.F. (2020). Facile and fast synthesis of SnO_2_ quantum dots for high performance solid-state asymmetric supercapacitor. J. Alloy. Compd..

[11] Chen S., Xing W., Duan J.J., Hu X.J., Qiao S.Z. (2013). Nanostructured morphology control for efficient supercapacitor electrodes. J. Mater. Chem. A.

[12] Xu B., Zhang H.B., Mei H., Sun D.F. (2020). Recent progress in metal-organic framework-based supercapacitor electrode materials. Coord. Chem. Rev..

[13] Pan Q.Q., Yang C., Jia Q., Qi W.T., Wei H.M., Wang M.X., Yang S.H., Cao B.Q. (2020). Oxygen-deficient BiFeO_3_-NC nanoflake anodes for flexible battery-supercapacitor hybrid devices with high voltage and long-term stability. Chem. Eng. J..

[14] Hwang S.W., Hyun S.H. (2007). Synthesis and characterization of tin oxide/carbon aerogel composite electrodes for electrochemical supercapacitors. J. Power Sources.

[15] Zhou K., Zhou W.J., Yang L.J., Lu J., Cheng S., Mai W.J., Tang Z.H., Li L.G., Chen S.W. (2015). Ultrahigh-performance pseudocapacitor electrodes based on transition metal phosphide nanosheets array via phosphorization: A general and effective approach. Adv. Funct. Mater..

[16] Kim S.C., Park Y.K., Kim B.H., An K.H., Lee H., Lee S.J., Jung S.C. (2017). Tin oxide/carbon nanocomposites as the electrode material for supercapacitors using a liquid phase plasma method. J. Nanosci. Nanotechnol..

[17] Wang H.K., Rogach A.L. (2013). Hierarchical SnO_2_ nanostructures: Recent advances in design, synthesis, and applications. Chem. Mat..

[18] Abdollahi H., Samkan M., Mohajerzadeh M.A., Sanaee Z., Mohajerzadeh S. (2019). High-performance tin-oxide supercapacitors using hydrazine functionalising assisted by hydrogen plasma treatment. Micro Nano Lett..

[19] Pusawale S.N., Deshmukh P.R., Lokhande C.D. (2011). Chemical synthesis of nanocrystalline SnO_2_ thin films for supercapacitor application. Appl. Surf. Sci..

[20] Asen P., Haghighi M., Shahrokhian S., Taghavinia N. (2019). One step synthesis of SnS_2_-SnO_2_ nano-heterostructured as an electrode material for supercapacitor applications. J. Alloy. Compd..

[21] Bonu V., Gupta B., Chandra S., Das A., Dhara S., Tyagi A.K. (2016). Electrochemical supercapacitor performance of SnO_2_ quantum dots. Electrochim. Acta.

[22] Mevada C., Mukhopadhyay M. (2020). High mass loading tin oxide-ruthenium oxide-based nanocomposite electrode for supercapacitor application. J. Energy Storage.

[23] Yadav A.A. (2016). Spray deposition of tin oxide thin films for supercapacitor applications: Effect of solution molarity. J. Mater. Sci. Mater. Electron..

[24] Selvan R.K., Perelshtein I., Perkas N., Gedanken A. (2008). Synthesis of hexagonal-shaped SnO_2_ nanocrystals and SnO_2_@C nanocomposites for electrochemical redox supercapacitors. J. Phys. Chem. C.

[25] Hong X.D., Li S.L., Wang R., Fu J.W. (2019). Hierarchical SnO_2_ nanoclusters wrapped functionalized carbonized cotton cloth for symmetrical supercapacitor. J. Alloy. Compd..

[26] Ramesh S., Yadav H.M., Lee Y.J., Hong G.W., Kathalingam A., Sivasamy A., Kim H.S., Kim H.S., Kim J.H. (2019). Porous materials of nitrogen doped graphene oxide@SnO_2_ electrode for capable supercapacitor application. Sci. Rep..

[27] Chuai M.Y., Chen X., Zhang K.W., Zhang J., Zhang M.Z. (2019). CuO-SnO_2_ reverse cubic heterojunctions as high-performance supercapacitor electrodes. J. Mater. Chem. A.

[28] Bhaskar V., Siddiqui M.J., Hakeem A.A. (2020). Synthesis of mesoporous SnO_2_/NiO nanocomposite using modified sol–gel method and its electrochemical performance as electrode material for supercapacitors. Sci. Rep..

[29] Rani B.J., Raj S.P., Ravi G., Yuvakkumar R. (2018). Surfactant free SnO_2_ nanoplate array synthesis for supercapacitor applications. AIP Publ. LLC.

[30] Wang Q., Tian Y., Guan S.D., Peng Z.J., Fu X.L. (2020). Alloying enhanced supercapacitor performance based on oxygen-deficient tin oxide nanorod array electrodes. ACS Appl. Energy Mater..

[31] Meng X.Q., Zhou M., Li X.L., Yao J.Y., Liu F.L., He H.C., Xiao P., Zhang Y.H. (2013). Synthesis of SnO_2_ nanoflowers and electrochemical properties of Ni/SnO_2_ nanoflowers in supercapacitor. Electrochim. Acta.

[32] Yerlanuly Y., Christy D., Van Nong N., Kondo H., Alpysbayeva B., Nemkayeva R., Kadyr M., Ramazanov T., Gabdullin M., Batryshev D. (2020). Synthesis of carbon nanowalls on the surface of nanoporous alumina membranes by RI-PECVD method. Appl. Surf. Sci..

[33] Dong Y.B., Liu F. (2020). Sodium dodecyl sulfate-assisted fabrication of NiO nanowalls grown on nickel foam as supercapacitor electrode materials. J. Mater. Sci. Mater. Electron..

[34] Choi H., Kwon S., Kang H., Kim J.H., Choi W. (2019). Adhesion-increased carbon nanowalls for the electrodes of energy storage systems. Energies.

[35] Bobadilla L.F., Romero-Sarria F., Centeno M.A., Odriozola J.A. (2016). Promoting effect of Sn on supported Ni catalyst during steam reforming of glycerol. Int. J. Hydrogen Energy.

[36] Xu L.Q.Y., Zhang L.Y., Cheng B., Yu J.G. (2019). Rationally designed hierarchical NiCo_2_O_4_-C@Ni(OH)_2_ core-shell nanofibers for high performance supercapacitors. Carbon.

[37] Wan L., Chen D.Q., Liu J.X., Zhang Y., Chen J., Xie M.J., Du C. (2020). Construction of FeNiP@CoNi-layered double hydroxide hybrid nanosheets on carbon cloth for high energy asymmetric supercapacitors. J. Power Sources.

[38] Wang Y., Chang Z., Qian M., Zhang Z.C., Lin J., Huang F.Q. (2019). Enhanced specific capacitance by a new dual redox-active electrolyte in activated carbon-based supercapacitors. Carbon.

[39] Ali A., Ammar M., Mukhtar A., Ahmed T., Ali M., Waqas M., Rasheed A. (2020). 3D NiO nanowires@NiO nanosheets core-shell structures grown on nickel foam for high performance supercapacitor electrode. J. Electroanal. Chem..

[40] Ratha S., Marri S.R., Lanzillo N.A., Moshkalev S., Nayak S.K., Behera J.N., Rout C.S. (2015). Supercapacitors based on patronite–reduced graphene oxide hybrids: Experimental and theoretical insights. J. Mater. Chem. A.

[41] Xie Y.B., Zhu F. (2017). Electrochemical capacitance performance of polyaniline/tin oxide nanorod array for supercapacitor. J. Solid State Electrochem..

[42] Zhu Y.H., Liu E.H., Luo Z.Y., Hu T.T., Liu T.T., Li Z.P., Zhao Q.L. (2014). A hydroquinone redox electrolyte for polyaniline/SnO_2_ supercapacitors. Electrochim. Acta.

[43] He G.J., Li J.M., Li W.Y., Li B., Noor N., Xu K.B., Hu J.Q., Parkin I.P. (2015). One pot synthesis of nickel foam supported self-assembly of NiWO_4_ and CoWO_4_ nanostructures that act as high performance electrochemical capacitor electrodes. J. Mater. Chem. A.

[44] Lu W., Yuan Z., Xu C.Y., Ning J.Q., Zhong Y.J., Zhang Z.Y., Hu Y. (2019). Construction of mesoporous Cu-doped Co_9_S_8_ rectangular nanotube arrays for high energy density all-solid-state asymmetric supercapacitors. J. Mater. Chem. A.

[45] Zhao J., Guan B., Hu B., Xu Z.Y., Wang D.W., Zhang H.H. (2017). Vulcanizing time controlled synthesis of NiS microflowers and its application in asymmetric supercapacitors. Electrochim. Acta.

[46] Chen F.S., Wang Z.N., Huo S.H., Ji S., Wang H., Zhou P.X. (2019). Cubic CoMn_2_O_4_ particles directly grown on Ni foam as binder-free electrode for asymmetric supercapacitors. Mater. Lett..

[47] Lu X.H., Zeng Y.X., Yu M.H., Zhai T., Liang C.L., Xie S.L., Balogun M.S., Tong Y.X. (2014). Oxygen-deficient hematite nanorods as high-performance and novel negative electrodes for flexible asymmetric supercapacitors. Adv. Mater..

[48] Du W.M., Zhu Z.Q., Xu Y.L., Zhang Z.H., Xiong X., Geng P.B., Pang H. (2015). High-performance asymmetric full-cell supercapacitors based on CoNi_2_S_4_ nanoparticles and activated carbon. J. Solid State Electrochem..

